# Effect of Denosumab and Teriparatide on Fracture Nonunion

**DOI:** 10.1210/jcemcr/luad107

**Published:** 2023-09-14

**Authors:** Regine Boutin, Sherri-Ann M Burnett-Bowie

**Affiliations:** Endocrine Division, Massachusetts General Hospital & Harvard Medical School, Boston, MA 02114, USA; Endocrine Division, Massachusetts General Hospital & Harvard Medical School, Boston, MA 02114, USA

**Keywords:** osteoporosis, fracture, nonunion, antiresorptive, teriparatide, denosumab

## Image Legend

A 59-year-old post-menopausal woman with class I obesity and type 2 diabetes mellitus complicated by nephropathy was referred to our practice for possible pharmacological management of a fracture complicated by nonunion; she had no history of prior fragility fracture. The patient had sustained a comminuted spiral humeral fracture after falling while walking her dog 5 months prior to endocrine consultation. At her 4-month post-fracture orthopedic follow-up, there was no bridging bone (Panel A). Dual x-ray absorptiometry showed osteopenia with posterior-anterior spine, total hip, and femoral neck T scores of −1.7, −1.5, and −1.8, respectively, and 28% bone mineral density loss since her dual x-ray absorptiometry from a decade ago. She was not taking medications that affected bone mineral density. Serum creatinine, calcium, 25-hydoxyvitamin D, and parathyroid hormone were normal. Given the nonunion and prior evidence from our group that denosumab and teriparatide combination therapy improves bone mineral density in post-menopausal women more than teriparatide alone [1], we initiated combination therapy. Imaging 7 and 13 months later (Panels B and C, respectively) revealed bridging bone and callus formation consistent with bone healing. These findings suggest that, like zoledronic acid and teriparatide combination therapy [2], denosumab and teriparatide combination therapy has the potential to promote healing in fractures complicated by nonunion.

**Figure:**
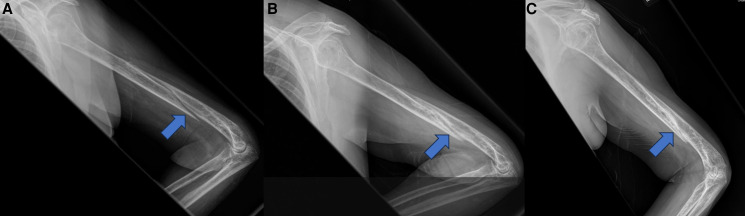


## Data Availability

Original data generated and analyzed during this study are included in this published article.
